# Metabolic Regulation Mechanisms of the Hypoglycemic and Anti‐Obesity Effects of *Ficus pumila* L. Var. *awkeotsang* Achene Extracts in 3T3‐L1 Cells

**DOI:** 10.1002/fsn3.70176

**Published:** 2025-04-15

**Authors:** Yu‐Siang Huang, Hsiao‐Ho Chen, Yuan‐Tay Shyu, Sz‐Jie Wu

**Affiliations:** ^1^ Department of Horticulture and Landscape Architecture National Taiwan University Taipei Taiwan; ^2^ Food Safety Center National Taiwan University Taipei Taiwan

**Keywords:** 3T3‐L1 differentiation, anti‐obesity, *Ficus pumila* L. var. *awkeotsang* extract, hypoglycemic effect, pectin

## Abstract

Jelly fig ( *Ficus pumila* L. var. *awkeotsang*) a species unique to Taiwan has been used for centuriesas sweets and snacks. It also has potential for medicinal purposes. In this study, the functional efficacy of the extracts of the achenes of different strains of jelly figs was compared. We found that the 80% methanol extract of the Hong‐jiou strain had a favorable inhibitory effect on the glycolytic enzymes. Furthermore, 3T3‐L1 cells were used to assess whether the extract of the Hong‐jiou strain can help regulate the transportation and utilization of glucose in the body and to investigate the insulin‐related signal transmission and regulation. According to the results of Oil Red O staining, the Hong‐jiou extract inhibited the formation of lipid droplets in both the prevention group and the curing group, and the determination of triglyceride content also showed that it reduced fat accumulation and the degree of differentiation. The three concentrations of the prevention group and the curing group revealed that the increase in glucose uptake was concentration dependent. Based on the comprehensive research results, the prevention group 200 μg·mL^−1^ (p‐200 group) was identified to have the greatest potential to inhibit obesity and improve hyperglycemia. According to the analysis of related protein expression based on the mRNA reverse transcription assay, the Hong‐jiou extract affected the mRNA expressions of PPARγ, SREBP‐1c, IRS1, LIPE, and CPT1 to reduce the degree of differentiation and the accumulation of fatty acids as well as increase glucose uptake, thereby having the potential hypoglycemic and anti‐obesity effects.

## Graphical abstract

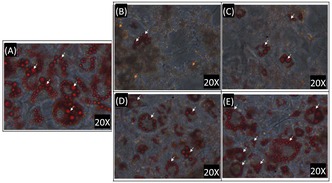

## 1 Introduction

Jelly fig ( *Ficus pumila* L. var. *awkeotsang*) a species unique to Taiwan, where jelly fig drink is a popular delicacy. While the medicinal functions of similar plants have been investigated in other countries, the medicinal use of jelly figs warrants more attention. Previous studies have reported the hypoglycemic effects of *Ficus pumila* Linn., which is a close relative species of *Ficus pumila* L. var. *awkeotsang*; *F. pumila* increases glycogen production in the liver and activates insulin signal transmission pathways. Moreover, it also reduces dietary fat absorption and increases plasma high‐density lipoprotein (HDL) cholesterol content to alleviate obesity and hyperlipidemia (Wu et al. 2017). The digestive enzymes α‐amylase and α‐glucosidase play pivotal roles in carbohydrate metabolism. α‐amylase is present in human saliva and the pancreas and can hydrolyze the α (1 → 4) glycosidic bond in starch into small molecules such as maltose or dextrin. Maltose or dextrin may be further decomposed into glucose by α‐glucosidase, which exists in the villus of the intestinal tract of animals and acts on the α (1 → 4) bond at the non‐reducing end of the sugar molecule to hydrolyze and produce glucose. The molecules that α‐glucosidase acts on include disaccharides, oligosaccharides, and large molecules of dextrin and starch. Long‐term studies have suggested that polyphenolic compounds have garnered attention for their ability to inhibit starch‐hydrolyzing enzymes and prevent the formation of advanced glycation end products, suggesting a role in diabetes management. Furthermore, polyphenolic compounds can increase the glucose transport signals produced by cells upon stimulation by insulin, which may improve hyperglycemia and directly reduce fat formation (Arulselvan et al. 2014; Dias et al. 2017). In addition, many studies have also shown that chemical substances in various plants may promote inflammation and fat catabolism, thereby increasing energy expenditure and inhibiting obesity‐related complications. Different components may affect the physiological metabolisms via different pathways (Cheung et al. 2016; Li et al. 2019).

The achenes and seeds of jelly fig are rich in various bioactive components. The achenes contain up to 42.35% dietary fiber, with protein and fat contents of 3.47% and 2.72%, respectively. Both the achenes and seeds are abundant in total flavonoids, and their fatty acid composition is dominated by α‐linolenic acid (62.65%), linoleic acid (18.24%), and oleic acid (10.62%), with unsaturated fatty acids accounting for 91.51%, higher than many commonly used vegetable oils (Yang et al. 2018).

Dietary fiber is categorized based on water solubility into soluble dietary fiber (SDF) and insoluble dietary fiber (IDF). Compared with IDF, SDF is highly fermented by coliform bacteria. Moreover, SDF is also regarded as a type of probiotic, which facilitates the growth of other probiotics, moderately increases stool volume, and produces short‐chain fatty acids (SCFAs) or secondary metabolites from microorganisms, thereby affecting the expression of many genes in the gut, which can affect digestive function, lipid and glucose metabolism, immune system, and cancer (Simpson and Campbell 2015; Musco et al. 2015; Comino et al. 2018). In addition to health benefits, low‐viscosity dietary fibers are generally used to change food texture and rheology, which can comprehensively affect the applicability of food systems. Moreover, these fibers can be used as health‐promoting products or functional products to increase the value of food in the market (Sharma et al. 2006; Gu et al. 2020). Pectin, a type of SDF, is an acidic heteropolysaccharide composed of galacturonic acid units that are polymerized via α‐(1 → 4)‐glycosidic bonds. Pectin is widely distributed in the cell wall of higher plants, including all land plants. Miyazaki et al. (2004) investigated the role of pectin in delaying drug release in jelly figs and investigated whether pectin of jelly figs can be used to prepare sustained‐release drug tablets by assessing its water absorption property (Miyazaki et al. 2004). Using theophylline sustained‐release tablets as a control, Miyazaki et al. (2004) reported that sustained‐release tablets prepared using jelly fig pectin exhibit a similar release curve, but they were not affected by pH. However, the degree of corrosion of the tablets was much slower, which was probably because pectin is a hydrophilic matrix and has favorable water absorption properties, resulting in the swelling of materials to form gels (Miyazaki et al. 2004). The study proposed that the unique “egg‐box structure” of the gel of jelly fig pectin likely (Komae et al. 1989) enabled the controlled drug delivery of tablets with a hydrophilic matrix (Miyazaki et al. 2004).

Numerous studies have shown that various plant‐derived chemicals can promote fat metabolism and anti‐inflammatory responses, thereby increasing energy expenditure and mitigating obesity‐related complications. These compounds include resveratrol, chlorogenic acid, curcumin, phytosterols, caffeine, catechins, flavonoids, and phenolic acids, each potentially affecting physiological metabolism through different pathways (Cheung et al. 2016; Li et al. 2019). For instance, the anti‐inflammatory and analgesic effects of methanolic extracts from *Ficus pumila* have been explored. These extracts have been identified to contain a variety of polyphenolic compounds, such as apigenin, luteolin, rutin, genistein, hesperidin, astragalin, isoquercitrin, and chrysin. Studies have found that apigenin and luteolin exert anti‐inflammatory effects by inhibiting ERK and JNK signaling pathways, reducing the production of pro‐inflammatory cytokines IL‐31 and IL‐33 in microglial cells (Chen et al. 2019). Additionally, rutin (23 μg/mL) and quercetin (6 μg/mL) can enhance AKT phosphorylation, promote GLUT‐2 translocation, and increase glucose uptake, thereby alleviating high‐glucose‐induced insulin resistance (Martín and Ramos 2021).

Lin et al. (2022) found that the jelly fig achene is rich in various nutrients and biologically active ingredients that are anti‐inflammatory and scavenging of free radicals (Lin et al. 2022). Moreover, it is non‐toxic and can also be used for cosmetic products. Chang (2008) extracted jelly fig powder using three solvents of different polarities and reported that the methanol extract has a better antioxidant effect, using which the DPPH free radical scavenging capacity reached 96% (Chang 2008). Moreover, the total phenolic content of the methanol extract was the highest at 320 mg/g gallic acid equivalent (Chang 2008). The methanol extract contains polyphenols and flavonoids, as revealed by high‐performance liquid chromatography. Recent studies have found that the polyphenolic compounds purified from jelly figs have high anti‐proliferation activity against U937 human monocytic leukemia cells as these compounds stagnate the cells in the G2/M phase of the cell cycle. Assessment of cell morphology has revealed that the polyphenol‐containing medium promotes the formation of apoptotic bodies, inhibits the growth by the immune stimulation of U937 leukemia cells, and also induces immature U937 cells to differentiate into mature leukocytes/macrophages (Shih et al. 2017). Based on these results, the present study aimed to investigate whether jelly fig extract can help regulate the digestion and absorption of glucose, fatty acids, and cholesterol in the gastrointestinal tract. We also explored the mechanisms of related extracts in the transmission and regulation of metabolic signals.

## 2 Materials and Methods

### 2.1 Extraction Method of Raw Materials

#### 2.1.1 Source of Test Samples

Wild type *Ficus pumila* L. var. *awkeotsang* achene (coded W), Hong‐jiou *Ficus pumila* L. var. *awkeotsang* achene (coded R), and Miao Li No. 2 *Ficus pumila* L. var. *awkeotsang* achene (coded M) were provided by the Taiwan Jelly Fig United Promotion Association. After drying, the jelly fig achenes of these three strains were sub‐packaged, vacuum‐sealed, and stored in a moisture‐proof box until right before the experiment when they were removed from the box.

#### 2.1.2 Preprocessing of Raw Materials

The fruits were first selected using a sieving machine with a sieve with 35 meshes, and then homogenized into powder by a Qiagen Tissuelyser II to be used as extraction raw materials.

#### 2.1.3 Extraction of Jelly Fig Achenes

The extraction of jelly fig achenes was performed by referring to the methods of Chang (2008). The dried jelly fig achenes were crushed into powder, and 0.5 g of this powder was weighed and extracted using deionized water and 80% methanol, respectively. A two‐stage vibration extraction method was adopted. After vibration at 1500 rpm for 15 min in each stage, the product was centrifuged at 18,600 *g* and 4°C for 10 min. Thereafter, the contents of total polyphenols and flavonoids of the crude extract were determined. A fixed volume of crude extract was placed in a microcentrifuge tube, concentrated, and dried with a centrifugal decompression concentrator at 35°C. The obtained dried substrate was redissolved and serially diluted for enzyme inhibition assays and cell experiments.

### 2.2 In Vitro Digestion Assay

#### 2.2.1 Analysis of Inhibition of α‐Glucosidase

For analysis of inhibition of α‐glucosidase, we referred to Kwon et al. (2007) and Tan et al. (2017) with slight modifications. Briefly, 50 μL of the extract and 50 μL of 0.1 M potassium phosphate buffer (pH 6.9 with 0.006 M NaCl) containing α‐glucosidase (1 U·mL^−1^) were pre‐reacted in a 96‐well plate at 25°C for 10 min. Thereafter, 0.1 M potassium phosphate buffer (pH 6.9) was added to prepare 50 μL of 5 mM p‐nitrophenyl‐α‐D‐maltoheptaoside solution. After mixing, the mixture reacted at 37°C for 30 min to measure the absorbance at 405 nm before and after the reaction. We used 50 μL of buffer in place of the extract to obtain the blank control group ($A_{405}^{\text{Blank}}$). The inhibition of α‐glucosidase was calculated as follows:

$$\%\text{inhibition}=\frac{∆A_{405}^{\text{Blank}}-∆A_{405}^{\text{Extract}}}{∆A_{405}^{\text{Blank}}}\times 100\%.$$

#### 2.2.2 Analysis of Inhibition of α‐Amylase

For analysis of inhibition of α‐ amylase, we used Kwon et al. (2007) and Sales et al. (2017) as references. We used porcine pancreatic α‐amylase (EC 3.2.1.1) for this process. Briefly, 100 μL of the extract and 100 μL of 0.02 M potassium phosphate buffer containing α‐amylase (2 U·mL^−1^) were pre‐reacted at 25°C for 10 min. Thereafter, 100 μL of 1% starch solution prepared with 0.02 M potassium phosphate buffer (pH 6.9 with 0.006 M NaCl) was added and reacted at 37°C for 10 min after mixing, and 200 μL of 3,5‐dinitrosalicylic acid (DNSA) was added and reacted in a boiling water bath to measure the absorbance value $A_{540\mathrm{nm}}^{\text{Extract}}$ at 540 nm. To determine the amount of reducing sugar originally contained in the extract, 100 μL of the extract and 200 μL of 0.02 M potassium phosphate buffer solution were mixed and allowed to stand for 20 min. The absorbance at 540 nm was measured using DNSA ($A_{540\mathrm{nm}}^{\text{Original}}$). The blank control group representing 100% enzyme activity was measured for the absorbance $A_{540\mathrm{nm}}^{\text{Blank}}$ at 540 nm using the same method.

The absorbance ($A_{540\mathrm{nm}}^{\text{Extract}}$) of the reducing sugar content generated by the decomposition of starch by α‐amylase was calculated as follows:

$$A_{540\mathrm{nm}}^{\text{Extract}}=A_{540\mathrm{nm}}^{\text{Test}}-A_{540\mathrm{nm}}^{\text{Original}}.$$

Calculation of the inhibition of α‐amylase:

$$\%\text{inhibition}=\frac{A_{540}^{\text{Blank}}-A_{540}^{\text{Extract}}}{A_{540}^{\text{Blank}}}\times 100\%.$$

### 2.3 Cell Experiments

#### 2.3.1 Cell Lines

In the present study, the mouse fibroblast 3T3‐L1 cell line was used, which was purchased from the Center for Biological Resource Conservation of the Food Industry Research and Development Institute. The cell line number was ATCC:CL‐173.

#### 2.3.2 Determination of Cell Viability Using MTS Assay

For determining cell viability, the MTS assay method described by Widowati et al. (2018) was used as a reference with modifications. Preadipocytes 3T3‐L1 were inoculated in a 96‐well plate at a density of 1 × 10^4^ cells·mL^−1^. After 24 h of culture, an equal amount of growth medium was replaced, and 50 μL of extracts at each concentration was added. The plates were placed in a 37°C incubator containing 5% CO_2_ and incubated for 24 and 48 h. The cell survival rate was determined using the MTS assay after replacing the culture medium with 130 μL of fresh DMEM. In addition to replacing the culture medium, 20 μL of CellTiter 96 AQueous One Solution reagent (Promega, USA) was added to each well. After incubation under the same conditions for 1 h, the absorbance at 490 nm was measured with an enzyme‐linked immunosorbent assay (ELISA) reader. The curing group in which no samples were added was used as the control group. It was used to compare the effects of the sample on the cell survival rate. Cell viability was expressed as a percentage using the following equation:

$$\text{Cell viability}\;\left(\%\right)=\frac{\text{Absorbance of sample group}}{\text{Absorbance of control group}}\times 100\%.$$

#### 2.3.3 3T3‐L1 Differentiation

To perform 3T3‐L1 differentiation, Lone et al. (2016) and Chen et al. (2018) were used as references with modifications. Briefly, 3T3‐L1 preadipocytes were inoculated in a 24‐well plate at a density of approximately 2 × 10^4^ cells per well. After culturing for 2 days, the medium was replaced to continue culture for cell confluency for 2 days. The old culture medium was removed and differentiation medium I, which contained differentiation inducing agents, was added to the culture for 72 h. Thereafter, the culture medium was replaced with differentiation medium II containing 1 μg·mL^−1^ of insulin. This culture medium was changed every 48 h to continue culture until the ninth day of differentiation. Mature adipocytes that were obtained using the differentiation method were used as the negative control (NC). In addition, the experimental groups were divided into prevention (p) and a curing (c) groups based on differentiation interventions. Subsequent cell analysis experiments were performed for each group on the ninth day of differentiation.

##### 2.3.3.1 Prevention Group

For differentiation intervention in this group, the jelly fig extracts at the prepared concentrations were added simultaneously along with the differentiation medium I or II, which was added to cells (from day 0 of differentiation).

##### 2.3.3.2 Curing Group

The differentiation intervention for the curing group included the addition of fig extract on the fifth and seventh days of the cell differentiation experiment.

#### 2.3.4 Oil Red O Staining

For Oil Red O staining, we referred to Lone et al. (2016) and Chen et al. (2018). Preadipocytes 3T3‐L1 were inoculated in 24‐well plates at a density of 2 × 10^4^ cells per well and induced into a negative control (NC), prevention (p), and a curing (c) groups, as per the aforementioned steps. After removing the medium, the cells were washed twice with PBS and then fixed with an appropriate amount of 10% formalin solution for 1 h. Thereafter, the solution was removed by sucking it out, and the cells were washed three times with PBS. The Oil Red O solution was diluted with sterile water at a ratio of 6:4, and the mixture was added to the cells for staining for 30 min. The cells were rinsed with sterile water after removing the staining agent and were observed and photographed under a microscope. Then the red stain was extracted with 100% isopropanol, and the absorbance at 490 nm was measured by using a spectrophotometer.

#### 2.3.5 Determination of Triglyceride (TG) Content

The study by Chen et al. (2018) was referred for determination of triglyceride (TG) content. Preadipocytes 3T3‐L1 were inoculated in 24‐well plates at a density of 2 × 10^4^ cells per well and induced into a negative control (NC), a prevention (p), and a curing (c) group, according to the aforementioned steps. The 3T3‐L1 adipocytes on the ninth day of differentiation were rinsed with PBS. Then 5% Triton X‐100 was added, and the mixture was allowed to stand for 5 min before cell lysis. The supernatant was collected into a microcentrifuge tube for 30 min of ultrasonic vibration. Thereafter, the product was centrifuged at 4°C and 12,000 *g* for 20 min to collect the supernatant. TG content was analyzed using the TG colorimetric assay kit.

#### 2.3.6 Glucose Uptake Assay

The glucose uptake assay was performed by following the experimental methods of Shen et al. (2014) and Mu et al. (2015) with modifications. The 3T3‐L1 cells in the 24‐well plates were induced to differentiate into a negative control (NC), prevention (p), and curing (c) groups according to the above steps. After removing the old medium of the 3T3‐L1 adipocytes on the ninth day of differentiation, the serum‐free no glucose DMEM growth medium was added, and the cells were cultured for 4 h to make it lack available glucose. The culture medium was removed, and the cells were washed three times with 0.5% BSA‐KRH buffer. Thereafter, the cells were cultured with 100 μL of no glucose DMEM, no glucose DMEM containing insulin (0.1 μM), no glucose DMEM containing apigenin (50 μM), and no glucose DMEM containing different concentrations of extracts for 20 min, respectively. Among them, insulin was used as the positive control drug, and apigenin was used as the negative control drug. Then 2‐NBDG (concentration per well was 100 μg·mL^−1^, equivalent to 300 μM) was added to co‐incubate for 20 min. Then, all the mixture was removed, and the reaction was terminated with PBS. The cell‐based assay buffer was used for replacement and rinsing. After centrifugation for removal, 100 μL of 5% Triton X‐100 was added, and the mixture was allowed to stand for 5 min for cell lysis. The supernatant was collected into a microcentrifuge tube for 30 min of ultrasonic vibration. Then centrifugation was performed at 4°C and 12,000 *g* for 20 min to collect the supernatant, which was diluted with cell‐based assay buffer at a ratio of 1:1. One hundred microlitre of the mixture was placed into a 96‐well black plate to measure the fluorescence value (excitation/emission = 475 nm/535 nm) with a multi‐mode microplate reader to calculate the glucose uptake.

### 2.4 Analysis of Related Protein Expressions Using mRNA Reverse Transcription Assay

#### 2.4.1 Total RNA Extraction From 3T3‐L1 Adipocytes

Total RNA extraction was performed using the protocol of the commercial kit Maxwell RSC simplyRNA Cells Kit. The 3T3‐L1 adipocytes on the ninth day of differentiation were rinsed twice with PBS, and then the attached cells were suspended with Trypsin–EDTA. The suspension was allowed to stand for 5 min, after which the serum‐containing growth medium was added to terminate the reaction, and the cell suspension was collected in Eppendorf tubes for subsequent use. The culture medium in the Eppendorf tubes was removed by centrifugation, and thereafter, 200 μL of 1‐thioglycerol/homogenization solution was added to each tube of cell samples. After 15 s of mixing with vortex, 200 μL of lysis buffer was added, and total RNA was extracted using a Maxwell RSC Instrument. NanoDrop was used to detect the content and quality of RNA.

#### 2.4.2 Polymerase Chain Reaction (PCR) for Reverse Transcription of RNA to cDNA

The commercial kit GoScriptTM Reverse Transcriptase (Promega, USA) was used for the polymerase chain reaction (PCR) for the reverse transcription of RNA to cDNA. Briefly, 1 μL of the purified RNA was added to the Reverse Transcription Reaction Mix reagent and subjected to PCR for reverse transcription. NanoDrop was used to detect the content and quality of cDNA, which was stored at −20°C. Subsequently, the obtained cDNA, adipogenic differentiation‐related gene primers (Table 1) (Guo et al. 2019; Hadrich and Sayadi 2018; Seo et al. 2019; Lingesh et al. 2019), and the GoTaq Green Master Mix reagent were mixed to perform PCR amplification under the conditions mentioned in Table 2.

**TABLE 1:** Primer sequences of adipose differentiation‐related gene.

Gene	Sequences	Citations
*Irs1*	5′‐AATAGCCGTGGTGATTACAT‐3′	Guo et al. (2019)
	5′‐CAGAAGCAGAAGCAGAGG‐3′	
*Srebf‐1c*	5′‐GCTTAGCCTCTACACCAACTGGC‐3′	Hadrich and Sayadi (2018)
	5′‐ACAGACTGGTACGGGCCACAAG‐3′	
*Pparγ*	5′‐AAACTCTGGGAGATTCTCCT‐3′	Hadrich and Sayadi (2018)
	5′‐TGGCATCTCTGTGTCAAC‐3′	
*Lipe*	5′‐CATTAGACAGCCGCCGTGCTG‐3′	Seo et al. (2019)
	5′‐TGGGGAGCTCCAGTCGGAAGA‐3′	
*Cpt1*	5′‐TGCCTCTGCCTTGATCTTTT‐3′	Lingesh et al. (2019)
	5′‐GGAACCGTGGATGAACCTAA‐3′	

**TABLE 2:** PCR amplification conditions.

	Step	Condition	Cycle number
*Irs1*	Initial denaturation	94°C, 15 min	1
	Amplification	94°C, 15 s 56°C, 60 s 72°C, 30 s	40
	Extension	72°C, 10 min	1
*Srebf‐1c* *Lipe*	Initial denaturation	95°C, 5 min	1
	Amplification	95°C, 30 s	35
		62°C, 30 s	
		72°C, 30 s	
	Extension	72°C, 7 min	1
*Parγ* *Cpt1*	Initial denaturation	95°C, 5 min	1
	Amplification	95°C, 30 s	40
		53°C, 30 s	
		72°C, 30 s	
	Extension	72°C, 7 min	1

#### 2.4.3 DNA Electrophoresis and Quantitative Analysis

PCR products were subjected to gel electrophoresis in an electrophoresis tank. The electrophoresis gel was prepared with 1X TAE buffer containing 1% agarose and 0.005% HealthyView. The size, quality, and integrity of the fragments were confirmed using PCR. Semi‐quantitative analysis of the brightness of the DNA bands on the gel was performed using the GeneTools 4.3.7 software.

### 2.5 Data Collation and Statistical Analysis

The statistical software XLSTAT was used for the analysis of variance (ANOVA). Tukey's honestly significant difference (HSD) test or Fisher's least significant difference (LSD) test was performed for post hoc testing, and a significance level of *p* < 0.05 was adopted. After statistical analysis of the chart data, the differences between different treatments were marked by lowercase a, b, c, d.

## 3 Results and Discussion

### 3.1 Analysis of Enzyme Inhibition Capacity

The capacity of the non‐gel extracts of jelly fig achenes to inhibit glycolytic enzymes was explored through in vitro experiments. We assessed whether the extracts affected the glycemic index (GI) by interfering with glucose production. The extracts were categorized as water extracts (coded W) and 80% methanol extracts (80 M). Table 3 shows that the half maximal inhibitory concentration (IC_50_) of Hong‐jiou water extract (RW) on α‐glucosidase was 67.55 ± 5.27 μg·mL^−1^, and the IC_50_ of Miao Li No. 2 water extract (MW) was 197.08 ± 4.27 μg·mL^−1^. In contrast, we were unable to estimate the IC_50_ of the water extract of the wildtype (WW) owing to its extremely low inhibitory capacity. In 80M extracts, the inhibitory capacity on α‐glucosidase was the highest in Hong‐jiou (R80M), followed by Miao Li No. 2 (M80M) and wild type (W80M), with IC_50_ of 5.37 ± 3.24, 18.87 ± 1.94, and 30.23 ± 3.45 μg·mL^−1^, respectively. This indicated that R80M extracts had the potential as an inhibitor of glycolytic enzymes. R80M and RW exhibited high inhibitory capacity to α‐amylase compared to the other two strains. Furthermore, no statistically significant differences were observed between IC_50_ values of RW and R80M. Hence, the extracts of the achenes of the Hong‐jiou strain have high potential to be an in vivo inhibitor of glycolytic enzymes.

**TABLE 3:** IC_50_ of Aiyuzi achene non‐gel extract extracted from different solvents on glycolytic enzyme.

Non‐gel extract	Half maximal inhibitory concentration (IC_50_) LS means	
	α‐amylase ($\mathrm{\mu g}∙{\mathrm{mL}}^{-1}$)	α‐glucosidases ($\mathrm{\mu g}∙{\mathrm{mL}}^{-1}$)
WW^1^, ^2^	220.83 ± 9.17^b^	> 1000^3^
W80M	42.05 ± 2.74^c^	30.23 ± 3.45^c^
MW	872.32 ± 164.43^a^	197.08 ± 4.27^a^
M80M	40.17 ± 2.23^c^	18.87 ± 1.94^d^
RW	19.40 ± 1.96^d^	67.55 ±5.27 ^b^
R80M	17.29 ± 3.92^d^	5.37 ± 3.24^e^

*Note:* All data are presented as the LS mean ± SD (*n* = 3). LS mean values followed by different letters in the same column indicate significant difference among three strain (*p* < 0.05, Tukey's test).

^1^ W‐wild cultivar; R‐Hong‐jiou cultivar; M‐Miaoli No. 2.

^2^ W‐water extraction; 80M‐80% methanol extraction.

^3^ > 1000: over detection limits.

Previous studies provide extensive references confirming that plant extracts can regulate carbohydrate metabolism and blood glucose control by inhibiting glycolytic enzymes. For example, Tan et al. (2017) demonstrated that phenolic compounds in black bean extracts exhibit excellent antioxidant capacity and significantly inhibit α‐glucosidase and lipase, showcasing diverse metabolic regulatory functions. Zhu et al. (2016) extracted a novel polysaccharide from Astragalus, which was experimentally proven to inhibit α‐glucosidase activity strongly. Additionally, Kerimi et al. (2017) reported that the polyphenol punicalagin in pomegranates is not only a highly effective α‐amylase inhibitor in vitro but may also regulate blood glucose levels by influencing glucose metabolism in intestinal and hepatic cells.

Bellesia et al. (2014) tested the inhibitory effects of pomegranate extract on α‐amylase and α‐glucosidase activities, finding strong in vitro inhibition of rat intestinal α‐glucosidase but relatively weaker effects on α‐amylase. Furthermore, results from simulated gastrointestinal digestion tests showed that pomegranate extract effectively reduced starch hydrolysis of potatoes during digestion, suggesting potential impacts on starch digestion efficiency in vivo (Bellesia et al. 2014). Although Chau et al. (2004) found that insoluble dietary fiber (IDF) from starfruit residues could lower blood glucose by adsorbing glucose, delaying its release, or impeding glycolytic enzyme activity, a comparison of Hong‐jiou methanolic extract (R80M) with starfruit residue showed that R80M exhibited stronger α‐glucosidase inhibition despite similar dietary fiber content. This suggests that phenolic compounds play a more critical role in glycolytic enzyme inhibition. The antioxidant properties of phenolic compounds may contribute to enzyme structural stability or interfere with enzyme activity, thereby enhancing inhibition.

These findings align with previous observations that R80M contains higher concentrations of polyphenols and flavonoids (Huang 2020), further supporting its remarkable effects in glycolytic enzyme inhibition. Based on these results, we propose that Hong‐jiou methanolic extract not only exhibits potent inhibitory capabilities but may also exert broader metabolic regulatory effects by modulating glucose transport and utilization in the body. Therefore, we further conducted 3T3‐L1 cell experiments using R80M extract to explore its potential impact on insulin signaling pathways, aiming to reveal its application value in diabetes and obesity management.

### 3.2 MTS Cell Viability and Cell Differentiation

**FIGURE 1:**
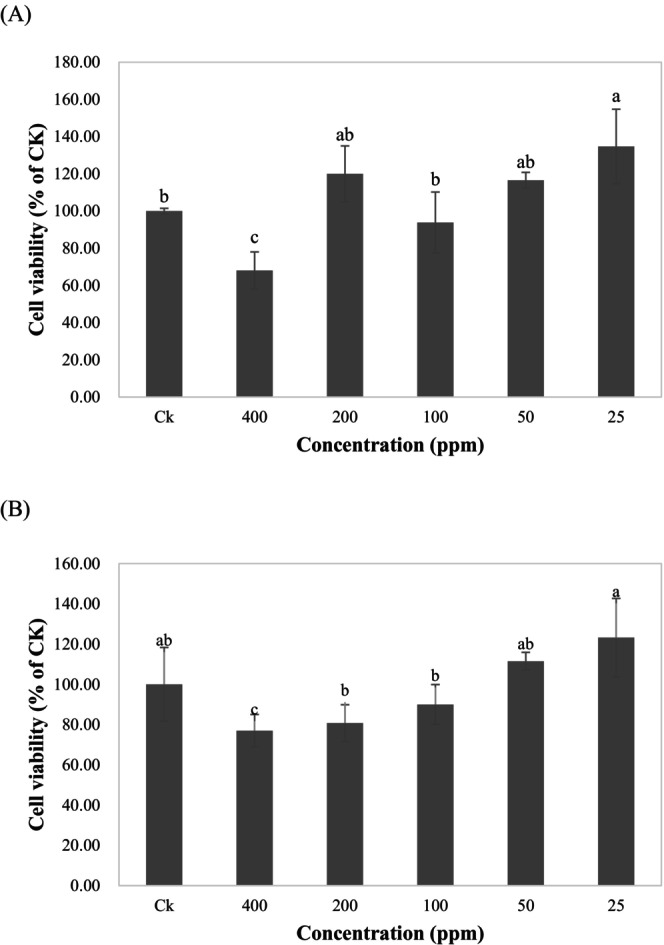
The cell survival rate of Hong‐jiou strain Aiyuzi achene non‐gel extract (R80M) and 3T3‐L1 cells co‐cultured for (A) 24 h and (B) 48 h. Ck: Control group (Ck) without the extract. All data are presented as the mean ± SD (*n* = 3). Mean values followed by different letters indicate significant difference among five group (*p* < 0.05, Fisher's test).

Figure 1A shows that during 24 h of co‐culture, the extracts at concentrations of 50, 100, 200, and 400 μg·mL^−1^ significantly inhibited cell viability, and the survival rate decreased by approximately 20%–40%. However, cell viability slightly increased in the 25 μg·mL^−1^ group compared to the control group (Ck) without the extract. This may likely be due to the increase in the dehydrogenase content in cell mitochondria within a short period of time after treating the cells with a small amount of extracts. This, in turn, could have enhanced the decomposition of MTS to generate more red‐purple formazan, thereby increasing the light absorption value. After 48 h of co‐culture (Figure 1B), a dose–response relationship between the concentration of each group and cell viability was observed, with a significant drop in cell viability in the 400 μg·mL^−1^ group. The cell viability was approximately 80%. No significant decrease in cell viability was observed in the 25, 50, 100, and 200 μg·mL^−1^ groups.

To be considered non‐toxic to cells, substances extracted from plants must produce a cell viability > 80% after treatment. However, the toxicity may gradually appear as the treatment time increases. According to the above experiment, for the treatment of 3T3‐L1 preadipocytes with the R80M extracts at 50, 100, and 200 μg·mL^−1^ for 48 h were chosen as the treatment concentrations for subsequent experiments.

**FIGURE 2:**
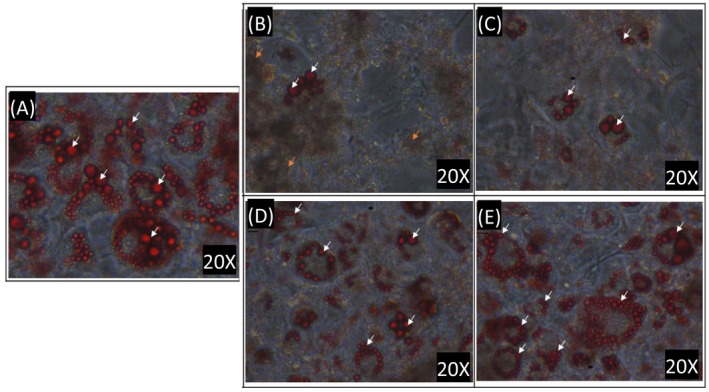
Micrographs of 3T3‐L1 adipocytes in different treatment groups induced to differentiate to oil red O staining on the ninth day. (A) Control group not co‐cultured with extract, (B) prevention‐200 ppm, (C) prevention‐100 ppm, (D) curing‐200 ppm, and (E) curing‐100 ppm. Fatty oil droplets are at the white arrow, apoptotic cells are at the orange arrow. Only electron micrographs showing the inhibition of lipid droplet formation are displayed.

**FIGURE 3:**
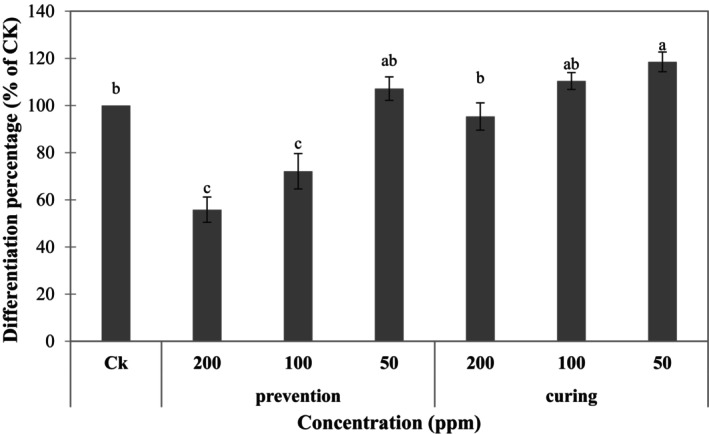
Oil red O staining to determine the differentiation rate of 3T3‐L1 adipocytes. CK: Control. All data are presented as the mean ± SD (*n* = 3). Mean values followed by different letters indicate significant difference among all group (*p* < 0.05, Tukey's test).

To determine whether the jelly fig extract had an anti‐obesity effect, we first assessed whether the extract inhibits the differentiation of preadipocytes and reduces fat accumulation. Oil Red O staining was performed 9 days after the induction of differentiation, and the obtained stain was used to assess whether there was accumulated fat in cells to obtain the degree of differentiation into adipocytes. The relative lipid content may be calculated through the dissolution of the dye for intergroup comparison. Figure 2 shows the microscopic observation results of cells treated on the 9th day of differentiation during the induction of 3T3‐L1 preadipocytes with the R80M extract at 200 and 100 μg·mL^−1^. (Only electron micrographs showing the inhibition of lipid droplet formation are displayed). Figure 3 shows that the R80M extract indeed inhibited the formation of fatty oil droplets. More than half of the cells in the control group that were not co‐cultured with the extract were stained, indicating a high differentiation rate and high lipid content in the cells. Significantly less TGs were stained in the prevention‐200 group and the prevention‐100 group. The fat content in the prevention‐50 group was similar to that of the control group, with no statistically significant differences. Therefore, it was speculated that the R80M extract had inhibitory effects on the differentiation and generation of fat cells as well as fat accumulation. Moreover, there was a concentration‐dependent effect for the inhibition. A concentration of 200 μg·mL^−1^ was required to produce an effect in the curing group provided in the middle of differentiation. The concentrations of the curing‐100 group failed to reduce the differentiation rate, and the fat content was not significantly different from that of the control group. The results of Tukey's HSD post hoc test showed no significant difference in the fat content between the curing‐200 group, curing‐100 group, and the control group. The fat droplets were only smaller when observed under the microscope, which may indicate that although the differentiation of adipocytes was not inhibited, these groups slowed down fat accumulation.

### 3.3 Determination of Triglyceride Content

**FIGURE 4:**
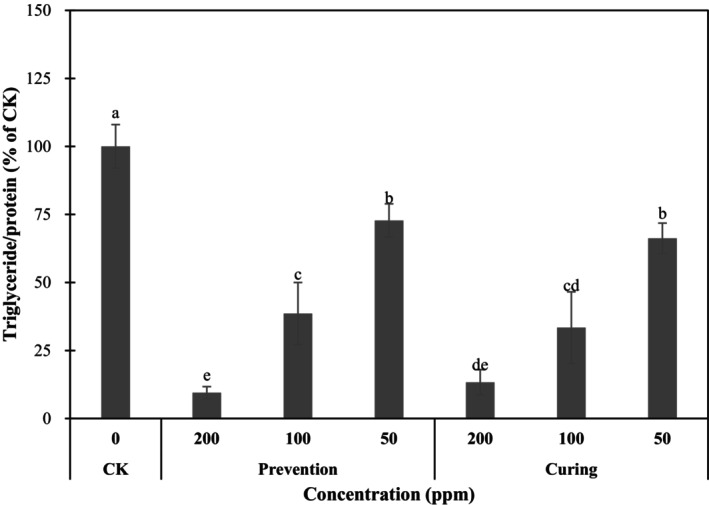
Effect of Hong‐jiou strain Aiyuzi achene non‐gel extract (R80M) on the accumulated triglyceride content of 3T3‐l1 pre‐adipocytes at the later differentiation stage. All data are presented as the mean ± SD (*n* = 3). Mean values followed by different letters indicate significant difference among all group (*p* < 0.05, Tukey's test).

The cells were collected and lysed on the ninth day of cell differentiation to analyze the TG content in the cells, as shown in Figure 4. The R80M extract was used to treat the cells. Compared with the TG content in the control group, the TG content in different prevention groups, 200, 100, and 50 μg·mL^−1^ groups, was inhibited by 90.49%, 61.39%, and 27.21%, respectively. Statistical analysis showed that TG contents at the three concentrations were all significantly different from that of the control group and that the inhibition exhibited a dose‐dependent effect. TG contents of the curing 200, 100, and 50 μg·mL^−1^ groups were inhibited by 86.43%, 66.85%, and 33.75%, respectively. Furthermore, the TG contents at the three concentrations were all significantly different from that of the control group and that the inhibition was dose dependent. Therefore, it was determined that treatment with the R80M extracts indeed reduced the TG contents and the degree of differentiation in cells. Concentrations of 200 and 100 μg·mL^−1^ may be the effective inhibitory concentrations. Moreover, inhibitory effects were seen in both the prevention groups and the curing groups. Meanwhile, according to the microscopic images, staining results, and TG determination results, the prevention group at 200 μg·mL^−1^ was the most effective anti‐obesity group. In the anti‐obesity studies using 3T3‐L1 adipocytes by Chen (2015), the reason why plant extracts inhibited adipocyte production may be related to the regulation of the protein expressions of PPARγ, C/EBPα, and UCP1. Ma et al. (2015) used chlorogenic acid to improve obesity and insulin sensitivity in mice fed a high‐fat diet. The study found that chlorogenic acid treatment of mice significantly reduced the total cholesterol in the liver as well as TG concentrations of obese animals. The change of mRNA expressions showed that fat synthesis signals such as FABP were downregulated, while the expressions of mRNA involved in fatty acid metabolism (such as CPT1, LIPE) were increased. In addition, it was also found that feeding chlorogenic acid reduced the inflammation‐related mRNA expressions of Pparγ1, Pparγ2, and MCP‐1. Therefore, the R80M extract was further co‐cultured with 3T3‐L1 to measure the mRNA expressions of related regulatory signals to understand the mechanisms of the inhibition of differentiation and fat accumulation.

### 3.4 Glucose Uptake Assay

**FIGURE 5:**
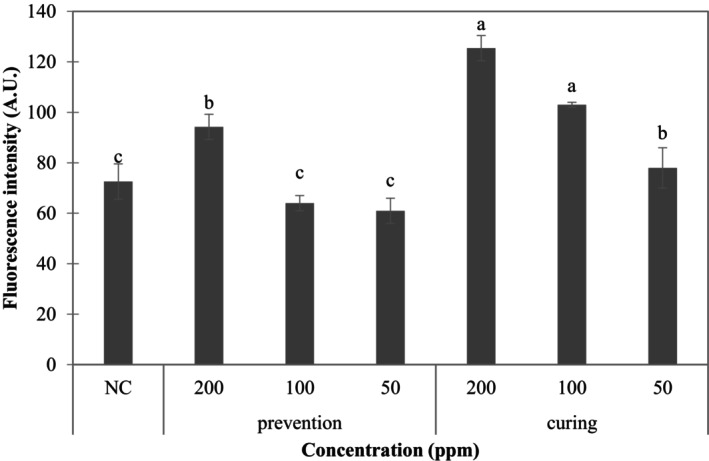
Effects of adipocytes in the prevention and treatment groups co‐cultured with extracts on the intake of 2‐NBDG. NC: Differentiation control group. All data are presented as the mean ± SD (*n* = 3). Mean values followed by different letters indicate significant difference among all group (*p* < 0.05, Tukey's test).

Figure 5 shows that after co‐culturing 3T3‐L1 cells with the R80M extract, higher concentrations of the extract increased glucose uptake. The three concentrations of the prevention groups and the curing groups show that the increase in glucose uptake was dependent on concentration. Moreover, the curing groups showed a significant increase in glucose uptake, with the uptake volume of the c‐200 group being not only higher than that of the p‐200 group but also almost twice that of the differentiated NC group. In a study by Hsu et al. (2021), ginsenosides exhibit a concentration‐dependent effect to increase the level of glucose uptake by mature adipocytes in the absence of insulin stimulation. When pretreated with insulin, part of the ginsenosides resulted in an uptake volume slightly lower than that of the control group. The authors speculated that glucose uptake by cells may decrease in the event of excessive stimulation. In the present study, cells in the prevention group were co‐cultured with the extract for 9 days. In contrast, the decrease may be caused by excessive stimulation. The degree of differentiation of the prevention group may likely be lower than that of the NC group. Therefore, in terms of the signal for glucose uptake, stimulation with extracts at higher concentrations was required to increase the uptake. Based on the result shown in Figure 5, it was speculated that the achene extract R80M could potentially assist in the regulation of blood glucose or regulate the signaling factors of glucose uptake by certain cells, which was further explored using mRNA reverse transcription assays.

### 3.5 Analysis of Related Protein Expressions Using mRNA Reverse Transcription Assay

**FIGURE 6:**
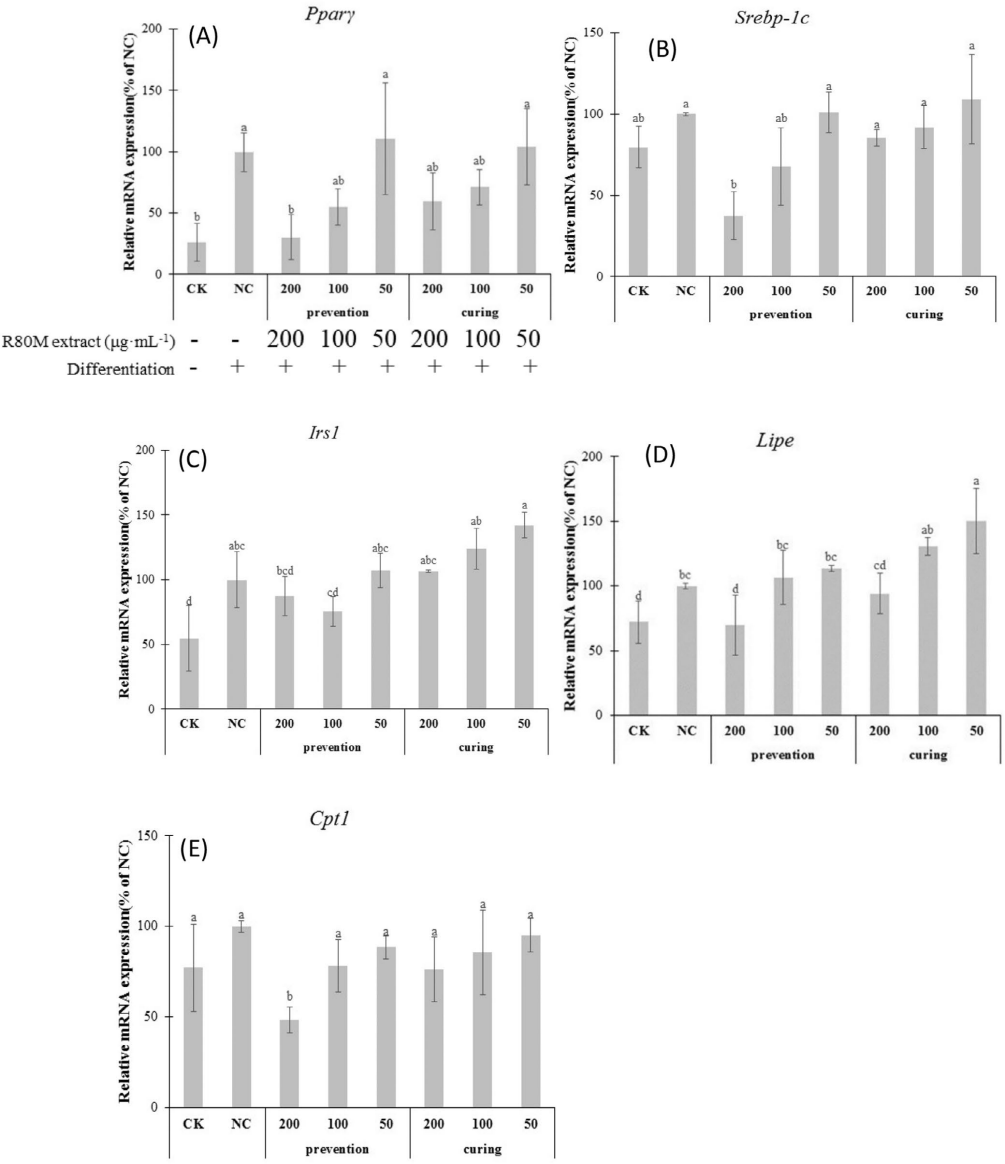
Effect of Hong‐jiou strain Aiyuzi achene non‐gel extract (R80M) on the differentiation and adipogenesis of 3T3‐L1 adipocytes pre‐adipocytes at the later differentiation stage. Measure the expression changes of (A) PPARγ, (B) SREBP‐1c, (C) IRS1, (D) LIPE, and (E) CPT1 through mRNA expression. CK, undifferentiated cell control group; NC, differentiation control group. All data are presented as the mean ± SD (*n* = 3). Mean values followed by different letters indicate significant difference among all group (*p* < 0.05, Tukey's test).

Figure 6A,B shows that PPARγ, which is closely related to inflammation and adipocyte differentiation, was significantly downregulated in the p‐200 group that received preventive treatment. In this case, the expression level was similar to that of the undifferentiated CK group. Slight decreases were also observed in the p‐100, c‐200, and c‐100 groups. Mature SREBP‐1c protein mainly regulates the signaling pathways required for fatty acid synthesis and for gene expression of FAS. Compared with the NC group, the expression of the SREBP‐1c protein in the p‐200 group reduced significantly. A slight downregulation was also observed in the p‐100 group. During the differentiation of NIH‐3T3 adipocytes, the expression level of SREBP‐1c is upregulated along with the differentiation time, which increases the expression levels of subsequent lipoprotein lipase (LPL) and FAS enzymes (Kim and Spiegelman 1996). In addition, PPARs are the key transcription factors in adipogenesis and are marker molecules of the early stage of adipocyte differentiation. The findings of the present study showed that R80M extracts reduced the level of differentiation as well as slowed down adipogenesis and inflammation by regulating the expressions of PPARγ and SREBP‐1c. This also explains the results of the differentiation rates and TG contents, which are discussed in previous sections. The prevention group 200 μg·mL^−1^ (p‐200 group) showed the highest potential for anti‐obesity activity.

Figure 6C shows that compared with the undifferentiated CK group, the IRS1 signal was slightly upregulated by R80M, but this change was not significantly dependent on concentration and the IRS1 expression increased more even at low concentrations. This finding was not completely consistent with the results of 2‐NBDG uptake (Figure 5), indicating that the R80M extract indeed affected IRS1 expression, which indirectly suggested that this extract increased the insulin sensitivity of adipocytes. Moreover, glucose uptake may be increased through changes in the expressions of other signaling regulatory factors, such as the activation of the AMP‐activated protein kinase (AMPK) (Kurimoto et al. 2013). Figure 6D,E shows that the R80M extract affected the expressions of LIPE and CPT1, which are factors related to lipolysis and β‐oxidation. When the cells were co‐cultured at a concentration of 200 μg·mL^−1^, the expressions of LIPE and CPT1 decreased due to the decrease in cell differentiation as well as the accumulation of fatty acids. In contrast, in groups with a higher degree of differentiation and the NC group, the mRNA expression (LIPE) of hormone‐sensitive lipase, the rate‐limiting enzyme for fatty acid decomposition, was upregulated. Further, significant increases were observed in the c‐100 and c‐50 groups. This indicated that the R80M extract accelerated the rate of fatty acid decomposition in differentiated and mature adipocytes. As seen in Figure 4, the TG contents of the prevention groups and the curing groups were lower than those of the control group.

Previous studies have found that soybean hull extract significantly reduces fasting plasma glucose and fasting plasma insulin in rats. Meanwhile, it also improved the physiological and biochemical indicators of obese and diabetic rats by regulating PPARγexpression (Tan et al. 2019). For example, chichoric acid, chlorogenic acid, caffeic acid, and cinnamic acid may improve insulin sensitivity, increase glucose uptake by cells, and upregulate the expressions of insulin signaling‐related proteins, including insulin receptors, PI3K, and glucose transporters (Huang et al. 2009; Ferrare et al. 2018; Chen et al. 2019). An overview of the findings of the present study of co‐culture of 3T3‐L1 and R80M extracts showed that the extracts reduced the TG contents and degree of differentiation. The prevention group at 200 μg·mL^−1^ was the most effective anti‐obesity group. Meanwhile, co‐culture at a concentration of 200 μg·mL^−1^ as both preventive and curing significantly increased glucose uptake. We speculated that a certain concentration of extracts may inhibit cell differentiation and fat accumulation by regulating the expression of insulin signaling‐related proteins, fatty acid synthesis, and metabolism‐related proteins. Notably, the increase in glucose uptake by cells did not result in the transformation and accumulation of fatty acids owing to the excess energy. This indicated that the promotion of glucose absorption from the environment by cells does not necessarily promote obesity, which is also related to other metabolic regulatory factors, such as those in the PPARs family. The PPARs family are important regulatory factors in processes such as energy balance, glucose metabolism, cholesterol and lipoprotein metabolism, fatty acid synthesis and oxidation, as well as cell proliferation. Drugs that activate PPARα and PPARγ have also been used to treat patients with diabetes mellitus (Wu et al. 1999; Rosen et al. 2002).

## 4 Conclusions

Extraction of Hong‐jiou strain (R80M) using 80% methanol extraction resulted in a strain with significantly high inhibitory activity on glycolytic enzymes compared to the strain obtained using water extracts (W). In previous studies that analyzed antioxidant capacity, the total polyphenol content, flavonoid content, reducing power, DPPH (1,1‐diphenyl‐2‐picryl‐hydrazyl) free radical scavenging capacity of R80M were higher than the values obtained for other groups. This suggests that R80M has high potential to inhibit glycolytic enzymes in vivo. In addition, after treating 3T3‐L1 cells with the R80M extract, the prevention group at 200 μg·mL^−1^ was the most effective anti‐obesity group based on the microscopic images and findings of staining and TG assay. Moreover, the analysis of related protein expressions based on the mRNA reverse transcription assay revealed that R80M extract indeed affected the mRNA expressions of PPARγ, SREBP‐1c, IRS1, LIPE, and CPT1. This reduced the degree of differentiation and the accumulation of fatty acids as well as increased glucose uptake, thereby potentially exerting hypoglycemic and anti‐obesity effects. Based on the results of cell experiments, future studies may explore the biochemical indicators and the performance of related proteins after feeding the extract to experimental animals. The findings of the present study will be helpful in the diet and health management of patients with diabetes, patients suffering from long‐term obesity, and the elderly. In addition, these results would improve the economic value of jelly fig achenes.

## Author Contributions

**Sz‐Jie Wu:** conceptualization (equal), funding acquisition (equal), methodology (equal), supervision (equal), validation (equal), writing – original draft (equal). **Yu‐Siang Huang:** data curation (equal), formal analysis (equal), investigation (equal), methodology (equal), software (equal), writing – original draft (equal). **Hsiao‐Ho Chen:** formal analysis (equal), investigation (equal), methodology (equal). **Yuan‐Tay Shyu:** conceptualization (equal), funding acquisition (equal), supervision (equal).

## Conflicts of Interest

The authors declare no conflicts of interest.

## Data Availability

The data that support the conclusions of this study are available upon request from the corresponding author.
